# Bring me the head of *Arthropleura*

**DOI:** 10.1126/sciadv.ads9192

**Published:** 2024-10-09

**Authors:** James C. Lamsdell

**Affiliations:** Department of Geology and Geography, West Virginia University, 98 Beechurst Avenue, Morgantown, WV 26505, USA.

## Abstract

After 170 years of searching, scientists finally know what the head of the largest known arthropod *Arthropleura* looked like, unlocking the key to its affinities.

Of all the creatures that once inhabited our planet, large arthropods are viewed with macabre fascination as eerie, almost otherworldly beings. From gargantuan dragonflies to colossal sea scorpions, the prospect of these chitinous creatures achieving such dimensions seems unnatural, as though their existence violates some fundamental law of nature. Nevertheless, during the mid- to late Paleozoic, arthropods attained sizes inconceivable among their modern descendants. Yet, even in a world of titanic bugs, *Arthropleura* stands out as the largest known arthropod that ever existed. Between 345 million and 290 million years ago, these creatures, more than 2.6 m long (*1*), crawled across what is today North America and Europe, leaving tread-like tracks in their wake (*2*, *3*).

Since its discovery in 1854, *Arthropleura *is one of the few extinct arthropods to be regularly featured in popular media, appearing in documentaries such as *Walking with Monsters* (2005), *First Life* (2010), and *Life on Our Planet* (2023) and the 2007 television series *Primeval.* The creature has also received attention as the subject of over 100 scientific papers. *Arthropleura*’s checkered taxonomic history features fierce debates about its affinities: Jordan and von Meyer described the first species as a decapod crustacean; Salter thought it was a type of eurypterid; Waterlot interpreted it as a distinct biramous arthropod equivalent in rank to Trilobita, while Manton considered *Arthropleura* a unique type of uniramian arthropod outside of either myriapods or insects, and Rolfe and Ingham assigned the genus to its own distinct group within myriapods. Most recently, Wilson and Shear (*4*) suggested that *Arthropleura* was a crown group millipede.

The reason for this uncertainty is simple: Despite the relative wealth of material written about it, *Arthropleura* is known from mostly fragmentary fossils. Characteristics of the creature’s head, for instance, were never known, confounding attempts to fully resolve its position within Myriapoda. Intimations that the head of *Arthropleura* was known but undescribed have persisted since the 1980s. However, the only publications describing the head were subsequently shown to mistakenly detail the collum, which is the body segment posterior to the head. Uncorroborated reports also suggested that *Arthropleura* possessed diplotergites—dorsal body sclerites that accommodated two pairs of limbs—as in millipedes. Ultimately, however, interpretations of *Arthropleura* have relied on comparison with more complete, closely related taxa. The generally accepted millipede identity for *Arthropleura* proposed by Wilson and Shear (*4*) is based on the anatomy of *Microdecemplex*, a much smaller creature from the Devonian.

The mystery of *Arthropleura* now appears solved. In this issue of *Science Advances*, Lhéritier *et al*. (*5*) describe exceptionally preserved specimens of *Arthropleura* from the Carboniferous of Montceau-les-Mines, France. Their remarkable findings, based on two almost complete juvenile individuals, present a new view of this enigmatic arthropod. With the aid of micro–computed tomography imaging, the authors unveil details of the fossils still buried in the rock matrix. The scans confirm that *Arthropleura* is undoubtedly a myriapod with affinities to the millipedes. A complete view of the animal’s posterior reveals the presence of a telson and demonstrates that the majority of the body segments were indeed diplotergites. However, the most exciting discovery comes from the specimens’ heads that bear a mosaic of millipede and centipede characteristics.

The seven-segmented antennae and mandibles with a distinct gnathal lobe are unquestionably those of a millipede, but the head lacks the plate-like gnathochilarium found in crown group millipedes. The presence of a modified collum behind the head is also typical of millipedes, but in *Arthropleura*, this manifests as a pair of limbs. In millipedes, the collum lacks appendages, indicating that *Arthropleura* cannot reside within the millipede crown group. However, the fully encapsulated mandibles and leg-like second maxillae seen in the new specimens are found in centipedes, although *Arthropleura* lacks the venomous forcipules characteristic of the group. The *Arthropleura* specimens also possess stalked compound eyes, features unknown among crown group myriapods but occurring in extinct euthycarcinoids which are known to resolve within the myriapod stem lineage (*6*). These details, together, may appear to leave *Arthropleura* as much—if not more—a puzzle than before but the seemingly chimeric nature of *Arthropleura* is actually important evidence that may help answer a fundamental question regarding the internal organization of Myriapoda.

Alongside centipedes and millipedes, two other groups of myriapods exist today: pauropods and pseudocentipedes, or garden centipedes. These two diminutive groups have long been considered to have close affinities to millipedes based on anatomical data and form a group called Progoneata, with centipedes distantly related to millipedes (*7*). This classic view was recently challenged by molecular data, which suggested a close relationship between centipedes and millipedes (forming a group called Pectinopoda) to the exclusion of pauropods and pseudocentipedes, which resolved in their own group called Edafopoda (*8*).

*Arthropleura*, combining millipede and centipede traits, may be the first morphological support for this molecular-based hypothesis of relationships. To test this, Lhéritier *et al*. conducted a phylogenetic analysis combining molecular and morphological data and included *Arthropleura* along with the purported arthropleurids *Eoarthropleura* and *Microdecemplex*. Their results support the uniting of centipedes and millipedes within Pectinopoda but do not retrieve arthropleurids as a natural group, showing that *Microdecemplex* is a crown group millipede, while *Arthropleura* resolves alongside *Eoarthropleura* either within the millipede stem group or within the stem group of Pectinopoda. The variability of the position of *Arthropleura* is entirely due to *Eoarthropleura*, which is poorly known with about 70% incomplete data in the analysis.

Lhéritier *et al*. suggest the incomplete picture may be causing *Eoarthropleura* to resolve more basally than its true phylogenetic position, effectively dragging *Arthropleura* with it. One key factor contributing to this is likely the uncertainty surrounding the condition of the limbs of *Eoarthropleura*, with Lhéritier *et al*. conservatively treating each tergite as accommodating only a single pair of limbs as in the original description of *Eoarthropleura* although subsequent authors have suggested *Eoarthropleura* to have diplotergites. In reality, the presence or absence of this critical millipede characteristic in *Eoarthropleura* is unknown. Overall, *Arthropleura* most likely represents a stem millipede (Fig. 1) and provides compelling morphological support for Pectinopoda, a result that has potential implications for our interpretations of other Paleozoic myriapod species. The position of *Arthropleura* also indicates that a number of centipede characteristics—such as the encapsulated mandibles and pediform second maxillae—represent the ancestral condition for Pectinopoda.

**Fig. 1.**
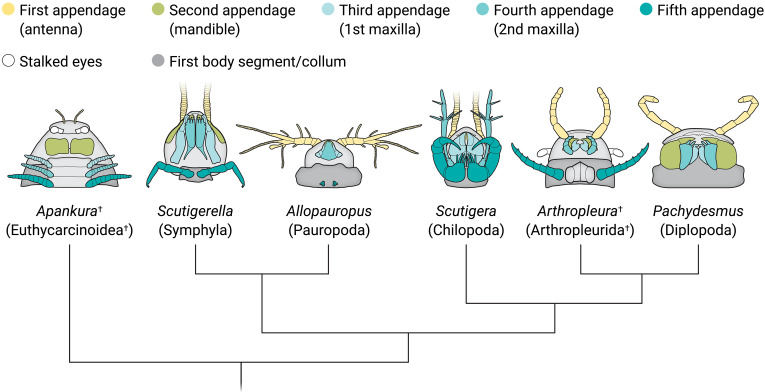
Phylogenetic position of *Arthropleura* among Myriapoda. The morphology and homology of head anatomy across euthycarcinoids, pauropods, symphylans, arthropleurids, chilopods, and diplopods are shown. Illustration credit: Ashley Mastin/*Science Advances.*

We now have, without a doubt, the most complete view of *Arthropleura* to date. Nevertheless, questions remain regarding this exceptional creature. On the basis of the head morphology and comparison with other myriapod taxa, the authors interpret *Arthropleura* as a detritivore (one who feeds on dead organisms); however, without direct evidence from digestive tracts, it is still unclear exactly what *Arthropleura* ate. The creature’s diet obviously has implications for its role in paleocommunities. It is of some interest given the large size the creature attained and that the development of large body size even in closely related arthropod groups likely had a variety of different causal factors (*9*). The respiratory organs also remain unknown, leaving the possibility that *Arthropleura* was aquatic (as previously suggested for *Eoarthropleura*). While the large tracks and diversity of environments where *Arthropleura* is found suggest a terrestrial life habit, the stalked eyes in the Montceau-les-Mines specimens (which are unlikely to represent an ancestral condition but instead an independently derived condition) are also known from euthycarcinoids which are likely amphibious (*10*). Stalked eyes may be an adaptation to a subaqueous lifestyle.

However, even if these specimens did have an amphibious habit, this does not mean that *Arthropleura* spent its entire life lurking around lakes and rivers. The specimens described by Lhéritier *et al*. are juveniles and as such afford a unique view into the ontogeny of this gigantic arthropod. The recognition of juvenile material has important ramifications for the delineation of species within the genus, as a number of previously identified *Arthropleura* species bear ornamentation closely resembling that of the juveniles and may themselves be juveniles. This is an important conclusion in and of itself, one likely to have major implications for our understanding of the species diversity and evolutionary history of *Arthropleura*. It is also possible that juveniles and adults inhabited slightly different environments and that the stalked eyes may have been a trait lost in adults. Future work evaluating the ontogenetic stage of *Arthropleura* species, as well as their environmental association, would likely be of great interest in determining whether juveniles occupied different habitats to adults. Looking to the future, it is likely that further discoveries regarding the biology of *Arthropleura* await. As the mystery of the affinities of the largest known arthropod is laid to rest, the work of reconstructing the life history of this exceptional creature can finally begin.

## References

[1] N. S. Davies, R. J. Garwood, W. J. McMahon, J. W. Schneider, A. P. Shillito, The largest arthropod in Earth history: Insights from newly discovered *Arthropleura* remains (Serpukhovian Stainmore Formation, Northumberland, England). J. Geol. Soc. 179, jgs2021-115 (2022).

[2] R. L. Martino, S. F. Greb, Walking trails of the giant terrestrial arthropod *Arthropleura* from the Upper Carboniferous of Kentucky. J. Paleo. 83, 140–146 (2009).

[3] J. O. Buckman, S. J. Cuthbert, P. G. Polson, *Arthropleura* trackway (*Diplichnites cuithensis*) from the Carboniferous, Serpukhovian, Limestone Coal Formation, Linn Park, Glasgow. Scott. J. Geol. 60, sjg2021-019 (2024).

[4] H. M. Wilson, W. A. Shear, Microdecemplicida, a new order of minute arthropleurideans (Arthropoda: Myriapoda) from the Devonian of New York State, USA. Trans. R. Soc. Edinb. Earth Sci. 90, 351–375 (2000).

[5] M. Lhéritier, G. D. Edgecombe, R. Garwood, A. Buisson, A. Gerbe, N. M. Koch, J. Vannier, G. Escarguel, J. Adrien, V. Fernandez, A. Bergeret-Medina, V. Perrier, Head anatomy and phylogenomics show the Carboniferous giant *Arthropleura* belonged to a milliped-centipede group. Sci. Adv. 10, eadp6362 (2024). 10.1126/sciadv.adp6362 PMC11463278 39383233

[6] G. D. Edgecombe, C. Strullu-Derrien, T. Góral, A. J. Hetherington, C. Thompson, M. Koch, Aquatic stem group myriapods close a gap between molecular divergence dates and the terrestrial fossil record. Proc. Natl. Acad. Sci. U.S.A. 117, 8966–8972 (2020). 32253305 10.1073/pnas.1920733117 PMC7183169

[7] W. A. Shear, G. D. Edgecombe, The geological record and phylogeny of the Myriapoda. Arthropod Struct. Dev. 39, 174–190 (2010). 19944188 10.1016/j.asd.2009.11.002

[8] L. R. Benavides, G. D. Edgecombe, G. Giribet, Re-evaluating and dating myriapod diversification and phylotranscriptomics under a regime of dense taxon sampling. Mol. Phylogenet. Evol. 178, 107621 (2023). 36116731 10.1016/j.ympev.2022.107621

[9] A. Ruebenstahl, N. Mongiardino Koch, J. C. Lamsdell, D. E. G. Briggs, Convergent evolution of giant size in eurypterids. Proc. R. Soc. B 291, 20241184 (2024). 10.1098/rspb.2024.1184 PMC11330558 39079669

[10] P. Gueriau, J. C. Lamsdell, R. A. Wogelius, P. L. Manning, V. M. Egerton, U. Bergmann, L. Bertrand, J. Denayer, A new Devonian euthycarcinoid reveals the use of different respiratory strategies during the marine-to-terrestrial transition in the myriapod lineage. R. Soc. Open Sci. 7, 201037 (2020). 33204464 10.1098/rsos.201037 PMC7657913

